# Modified R-GLIM Score Is a Good Prognostic Tool to Predict a Long-Term Prognosis in Poor Conditioned Elderly Patients with Aspiration Pneumonia, a Pilot Study

**DOI:** 10.3390/geriatrics9050118

**Published:** 2024-09-12

**Authors:** Yoshinori Wakita, Nobuhiro Asai, Wataru Ohashi, Naoharu Mori, Masato Maekawa, Hiroshige Mikamo

**Affiliations:** 1 Division of General Medicine, Aichi Medical University, Nagakute 480-1195, Aichi, Japan; 2 Department of Clinical Infectious Diseases, Aichi Medical University, Nagakute 480-1195, Aichi, Japan; 3 Division of Biostatistics, Clinical Research Center, Aichi Medical University, Nagakute 480-1195, Aichi, Japan; 4 Department of Palliative and Supportive Medicine, Graduate School of Medicine, Aichi Medical University, Nagakute 480-1195, Aichi, Japan

**Keywords:** malnutrition, high respiratory rate, modified GLIM criteria, aspiration pneumonia

## Abstract

Background. While prognostic guidelines for pneumonia have widely allowed clinicians to treat patients, poor prognostic factors for 1- or 2-year survival times have never been mentioned to our knowledge. Patients and methods. We conducted this retrospective study to evaluate whether malnutrition according to the GLIM criteria is a poor prognostic factor for 1- or 2-year survival among patients with aspiration pneumonia. All patients with community-onset aspiration pneumonia who were admitted to Aichi Medical University and had intervention from our nutrition support team (NST) in 2019 and 2020 were enrolled in this study. Results. A total of 56 patients were enrolled in the study. The mean age was 86 ± 6.5 and 25 (45%) were male. Thirty-one patients died during this observational period. Comparing the survival and death group, higher respiratory rate (RR) and malnutrition were seen more frequently in the death group than in the survival group. Then, the patients were divided into the following three groups: those with an RR ≥ 22 and malnutrition, those with malnutrition, and a control group [patients who were not malnourished and had a low RR (<22)]. Comparing the three groups, patients with an RR ≥ 22 and malnutrition had significantly shorter overall survival times (OSs) than those in the other groups (*p* = 0.009 by *Log-Rank* test) for 1-year prognosis. The result of 2-year prognosis displayed a statistical significance that was the same as that for 1-year prognosis (*p* = 0.004 by *Log-Rank* test). The Cox hazard regression model showed that a higher RR was an independent poor prognostic factor for 1- and 2-year survival among aspiration pneumonia patients. Conclusions. This pilot study showed that combined scores of higher RR and malnutrition according to the GLIM criteria (modified R-GLIM score) was an independent poor prognostic factor for 1 or 2-year survival among super-elderly patients (aged over 80 years) with aspiration pneumonia.

## 1. Introduction

Despite the advances in diagnostic methods and antibiotic therapies, pneumonia is the leading cause of death in the elderly worldwide [1,2]. Pneumonia is one of the most common diseases every physician has to face. Therefore, the prognostic guideline for pneumonia is essential for physicians to see patients, particularly for non-specialists. We previously reported that the prognostic guidelines for pneumonia in adults issued by the Japanese Respiratory Society (JRS) showed the efficacy and validity of the quick Sequential Organ Failure Assessment (qSOFA) and SOFA scores to predict the prognosis of patients with community-onset pneumonia [2,3]. Recent reports documented that qSOFA is not a valuable tool in evaluating the prognosis of infection [4,5], as it does not have enough predictive power. While qSOFA works to screen the severity of infection, it does not serve as a reliable tool in evaluating the prognosis. Several prognostic guidelines, A-DROP [6], CURB-65 [7], Pneumonia severity index (PSI) [8], and I-ROAD [9,10] have been issued and widely used to treat patients with pneumonia. A-DROP, CURB-65, and PSI are scoring tools used to evaluate the severity and prognosis of CAP. Only I-ROAD is used as a scoring tool to predict the severity and prognosis of hospital-associated pneumonia (HAP), which is defined as pneumonia when it develops after at least 48 h of being in hospital. While all of these are useful to evaluate the short-term prognosis of pneumonia, such as 30-day mortality, their usefulness for evaluating a long-term prognosis is unknown.

The Global Leadership Initiative on Malnutrition (GLIM) criteria, issued in 2018 by the Parenteral and Enteral Nutrition Society, is a diagnostic tool for diagnosing malnutrition in adults [11]. Some demonstrated that malnutrition evaluated by the GLIM criteria could be a poor prognostic factor among patients with chronic kidney and autoimmune diseases [12,13,14]. Thus, we hypothesized that malnutrition was associated with poor prognosis among pneumonia patients.

This pilot study assessed the new predictive score using a combined score with the respiratory rate (RR) and GLIM criteria for evaluating poor-conditioned healthcare-associated pneumonia (HCAP) patients instead of the qSOFA score. This is the first report documenting that the combined score of RR and the GLIM criteria, which is defined as the modified R-GLIM score, could predict a long-term prognosis (1- or 2-year survival times) among poorly conditioned elderly patients with aspiration pneumonia.

## 2. Patients and Methods

### 2.1. Study Design

All patients with community-onset aspiration pneumonia admitted to our institute were reviewed and evaluated by our nutrition support team (NST) in 2019 and 2020. After discharge from our hospital, we followed all patients using their medical charts. We contacted patients whose medical charts we needed to follow. Our institute, which has 900 beds, is a tertiary and educational hospital located in the countryside in Japan [2,15].

Patients’ characteristics, clinical symptoms, laboratory data, microbial testing results, radiological findings, initial antibiotic regimens used, and outcomes were examined. To identify a prognostic factor, we compared patients’ characteristics, clinical data, and microbial results between the two groups (survival and death groups). Then, the Cox proportional hazard regression analysis was carried out to find independently poor prognostic factors among the patients for 1- or 2-year survival.

### 2.2. Patients Enrolled

We enrolled the patients who met all the following criteria in the study. Hospital-acquired pneumonia (HAP) was excluded in the study. Those whom we could not follow were excluded from the study.

1. The onset of pneumonia was community-acquired pneumonia (CAP) or HCAP.
2. Those who had an intervention by the NST.
3. Those whom we could adequately follow the clinical course and prognosis of after discharge.

Aspiration pneumonia was diagnosed based on relevant clinical symptoms with radiological findings by chest-computed tomography, according to the previous reports [15]. All radiological images were assessed by independent expert radiologists and pulmonologists with more than 15 years of career experience.

### 2.3. Disease Severity and Nutritional Status

The severity of pneumonia was evaluated by A-DROP [6], CURB-65 [7], PSI [8], I-ROAD [9,10], SIRS [16], qSOFA, and SOFA score [17]. We used the Charlson Comorbidity Index (CCI) score to evaluate patients’ comorbidity [18,19]. The GLIM criteria assessed the nutritional status of the patients [11].

### 2.4. The Global Leadership Initiative on Malnutrition (GLIM) Criteria

The criteria consists of three phenotypic criteria (non-subjective weight loss, low BMI, and reduced muscle mass) and two etiologic criteria (reduced intake or digestive malabsorption, inflammation, or disease burden). Malnutrition was defined as meeting at least one of the phenotypic and one of the etiologic criteria [11].

### 2.5. Microbial Assessment

The antimicrobial susceptibility testing was performed according to the Clinical and Laboratory Standards Institute guidelines, and the antimicrobial susceptibility of isolated bacterial pathogens was assessed based on the minimum inhibitory concentrations [20]. Methicillin-resistant *Staphylococcus aureus*, *Pseudomonas aeruginosa*, *Acinetobacter baumannii*, and extended-spectrum β-lactamase-producing organisms were defined as potentially drug-resistant (PDR) pathogens based on ATS/IDSA guidelines and the previous studies [2,15,21].

### 2.6. Definition of Appropriate or Inappropriate Treatment and Initial Treatment Failure

According to the previous reports, antibiotic treatment was classified as appropriate or inappropriate according to whether the identified pathogens were susceptible or resistant to the initially prescribed antibiotics [2,15,22]. Initial treatment failure was defined as death during the initial treatment or a change in the antibiotic regimen from the initial agents within 72 h after starting the treatment due to a lack of response or clinical deterioration (e.g., worsening of fever, respiratory condition, or radiologic status; requiring mechanical ventilation, aggressive fluid resuscitation, or vasopressors) [2,15,22].

### 2.7. Statistical Analyses

The data for categorical variables are expressed as percentages and continuous variables as mean ± standard deviation (SD). Chi-square or Fisher’s exact test (two-tailed) was used to compare categorical variables and unpaired Student’s t-test or Mann–Whitney U test to compare continuous variables. Statistical analyses involved the use of SPSS version 26 for Windows (SPSS Inc., Chicago, IL, USA). Kaplan–Meier analyses were carried out using Graph Pad Prism v 9.3.1. Overall survival time (OS) was calculated from the date of diagnosis until the date of death from any cause. The generalized Wilcoxon test and Log-Rank test evaluated significance. We identified poor prognostic factors by comparing between the survival and death groups using a *p*-value < 0.1. Then, the Cox proportional hazard regression analysis showed independent poor prognostic factors among the patients. A *p*-value < 0.05 was considered statistically significant. This study was approved by the Institutional Review Board of Aichi Medical University Hospital (the IRB number 2022-123).

## 3. Results

Table 1 shows the patients’ profiles and clinical outcomes for 1-year prognosis. A total of 56 patients were enrolled in this study. The mean age was 86 [±standard deviation (SD) 6.5], and 25 were male (45%). All patients had HCAP. The most common underlying factors of HCAP was poor ADL requiring any physical support in 49 (88%), followed by nursing home residence in 30 (54%), as shown in Supplemental File Table S1. Concerning nutritional status, 48 (86%) were categorized into malnutrition according to the GLIM criteria. In terms of the severity of pneumonia, mean points of A-DROP, CURB-65, PSI, I-ROAD, SIRS, qSOFA, and SOFA score were 2.9 (±0.9), 2.6 (±1.0), 144.9 (±28.7), 2.8 (±0.9), 1.6 (±1.2), 1.4 (±0.7), and 3.2 (±1.9), respectively. The most common underlying diseases were dementia in 37 (66%), followed by cerebrovascular diseases in 30 (54%). The mean CCI was 2.6 (±1.4). As for patients’ conditions, higher respiratory rate (>22), shock, and bacteremia were seen in 24 (43%), 8 (14%), and 50 (89%) respectively.

Forty-three patients (77%) had sputum cultures at the initial visits. The most frequent pathogen was methicillin-susceptible *Staphylococcus aureus* in 10 (18%), followed by *Klebsiella pneumonia* in 8 (14%). PDR pathogens were isolated in 14 (25%). There was no significant difference in microbial isolation rate between the two groups (Supplemental File Table S2).

### 3.1. Comparison of Patients’ Profiles and Conditions between Survival and Death Group

We compared with the survival and death group to find a prognostic factor for 1-year prognosis. There were no differences in age, male gender ratio, or underlying diseases, including mean CCI scores. A higher RR and malnutritional status were more frequently found in the death group than in survival group.

#### 3.1.1. Comparison of Overall Survival Times for 1-Year Prognosis between Patients with Normal Nutritional Status or Malnutrition According to the GLIM Criteria 

**Figure 1:**
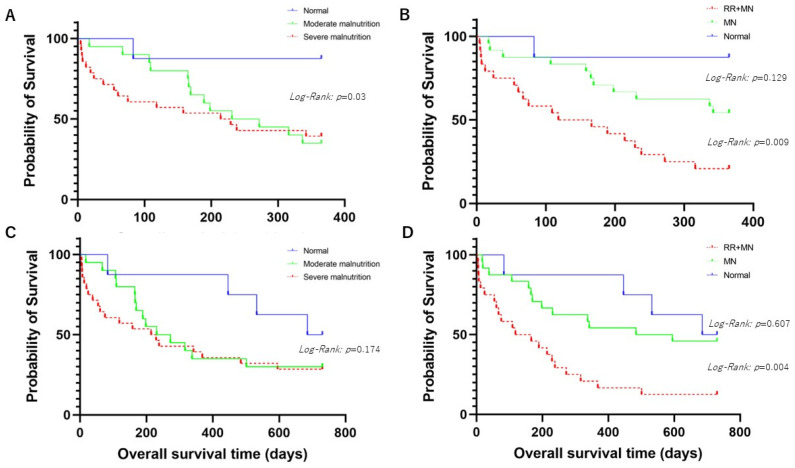
(**A**) shows comparison of overall survival time (OS) among aspiration pneumonia patients with mild malnutrition, moderate malnutrition, or normal nutrition status for 1-year prognosis. (**B**) shows comparison of OS among aspiration pneumonia patients with higher respiratory rate (RR) (≥22) and malnutrition (MN), MN, or normal nutrition status for 1-year prognosis. (**C**) shows comparison of OS among aspiration pneumonia patients with mild malnutrition, moderate malnutrition, or normal nutrition status for 2-year prognosis. (**D**) shows comparison of OS among aspiration pneumonia patients with higher RR (≥22) and MN, MN, or normal nutrition status for 2-year prognosis.

We divided the patients into the following three groups: normal or moderate malnutrition or severe malnutrition groups according to the GLIM criteria. In comparing the three groups, patients with normal nutritional status had significantly longer OSs than those in the other two groups (*p* = 0.03 by *Log-Rank* test) (Figure 1A). The patients were categorized into the following three groups: the patients with RR (≥22) and malnutritional status, those with malnutritional status, and the control group [those with a lower RR (<22) without malnutrition]. Comparing the three groups, those with a higher RR and malnutrition displayed significantly shorter OSs than those in the other groups (*p* = 0.009 by *Log-Rank* test) (Figure 1B).

#### 3.1.2. Comparison of Overall Survival Times for 2-Year Prognosis between Patients with Normal Nutritional Status or Malnutrition According to the GLIM Criteria

As above, the patients were classified into three groups. The patients with normal nutritional status tended to have a longer OS than those in the other two groups without statistical significance (*p* = 0.174 by *Log-Rank* test) (Figure 1C). Comparing the three groups by RR and the GLIM criteria, the patients with a higher RR and malnutrition had a significantly shorter OS than those in the other two groups (*p* = 0.004 by *Log-Rank* test) (Figure 1D). 

### 3.2. Receiver Operating Characteristic (ROC) Curves of Combined Score of RR and GLIM Criteria, the GLIM Criteria, A-DROP, CURB-65, PSI, I-ROAD, qSOFA, and SOFA Score for 1- and 2-Year Death

Supplemental Files Tables S3 and S4 show the result of the area under the receiver operating characteristic (AUROC) of each nutritional index. We defined the modified R-GLIM score as shown in Table 2. A high RR (≥22) and normal RR (<22) were scored as 1, 0. Nutritional status according to the GLIM criteria was scored as follows: normal nutritional status, moderate malnutrition, and severe malnutrition were 0, 1, and 2, respectively. The modified R-GLIM score ranged from 0 to 3. The AUROCs of combined-score RR and GLIM criteria (modified R-GLIM score) for 1- and 2-year death were 0.708 (95% CI 0.573–0.843, *p* = 0.008) and 0.669 (95% CI 0.525–0.822, *p* = 0.043), respectively (Table 3).

The prognostic accuracies of modified R-GLIM score are shown in Table 2. The appropriate cut-off value was 3 and had sensitivity of 41.9%, specificity of 92.3%, a positive predictive value of 86.7%, and a negative predictive value of 43.9% based on the Youden Index [23].

**Figure 2:**
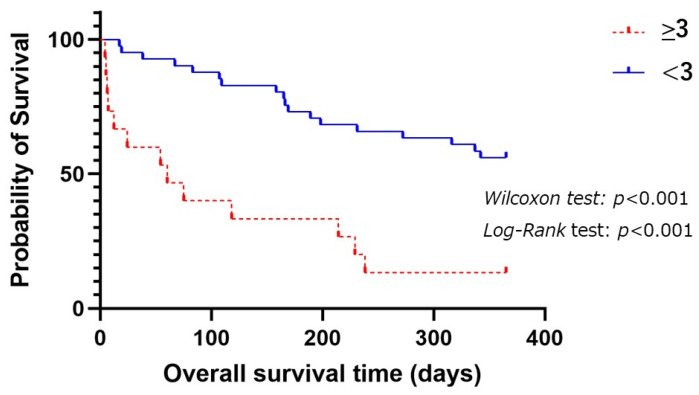
Comparison of OSs among aspiration pneumonia patients with a modified R-GLIM score ≥ 3 and <3 for 1-year prognosis.

Figure 2 shows comparison of OSs between a modified R-GLIM score ≥ 3 and <3 among the patients as follows. The patients with a modified R-GLIM score ≥ 3 had significantly shorter OSs than those with a modified R-GLIM score < 3 (*p* < 0.001 by *Log-Rank* test).

#### 3.2.1. Prognostic Factors for 1-Year Survival among Aspiration Pneumonia

We analyzed poor prognostic factors for 1-year survival among the patients. A higher RR (≥22) and malnutrition, according to the GLIM criteria, were poor prognostic factors for 1-year survival by univariate analysis. Of these two, the Cox hazard regression model showed that a higher RR (≥22) was an independent poor prognostic factor for 1-year survival among the patients, as shown in Table 4.

#### 3.2.2. Prognostic Factors for 2-Year Survival among Aspiration Pneumonia Patients

We analyzed poor prognostic factors for 2-year survival among the patients. A higher RR (≥22) was a poor prognostic factor for 2-year survival. The Cox hazard regression model showed that a higher RR (>22) was an independent poor prognostic factor for 2-year survival among the patients, as shown in Table 5. 

## 4. Discussion

Our study suggests that combined higher RR and malnutrition according to the GLIM criteria was an independent poor prognostic factor for one- or two-year survival among super-elderly (aged over 80 years) patients with aspiration pneumonia. Conventional prognostic guidelines help predict 30-day mortality rates but not for long-term survival. Kodama et al. reported that A-DROP could not correctly evaluate the prognosis among super-elderly patients with pneumonia [24]. One possible explanation is that respiratory symptoms are hard to be evaluated objectively, leading to underestimation of the severity of the pneumonia. As for the utility of the GLIM criteria to predict aspiration pneumonia, the GLIM score with the cut-off of 2 had sensitivity and specificity of 55 and 56% for 1-year survival and 53% and 56% for 2-year survival, which are lower than those of the modified R-GLIM score. Previous studies have documented that the RR change is likely to occur much faster than that of SpO_2_ decline [25]. Since RR has been considered to be a crucial prognostic factor among those with pneumonia, impacting the prognosis of pneumonia, it is reasonable that RR is used as a common denominator in CURB-65, PSI, and qSOFA. Due to these reasons, combined score RR and GLIM criteria (modified R-GLIM score) showed a higher prognostic accuracy for 1-and 2-year survival among the patients than the GLIM criteria alone, as shown in Supplemental Files Tables S3 and S4.

As shown in Figure 1, the group with malnutrition assessed by the GLIM criteria had shorter OSs than those without, even if malnutrition was not an independent poor prognostic factor. Malnutrition influences irregular immune response, resulting in poor prognosis in patients with aspiration pneumonia [26,27]. Some demonstrated that dysphagia correlated with nutrition status in the elderly [28]. Aspiration pneumonia induces muscle atrophy in the respiratory, skeletal, and swallowing systems via the expression of pro-inflammatory biomarkers such as C-reactive protein and IL-6. Consequently, diaphragmatic atrophy weakens the force of cough to expectorate sputum or mis-swallowed contents, resulting in sarcopenia [29]. There might be a vicious cycle of aspiration pneumonia based on a correlation between malnutrition and sarcopenia. In the study, 48 patients (85%) had sarcopenia according to the Asian Working Group for Sarcopenia 2019 criteria [30]. IJmker-Heninket, et al. reported that malnutrition according to the GLIM was a poor prognostic factor as well as an indicator for 1-year survival among hospitalized patients [31]. Trollebø MA et al. conducted a matched cohort study in Norway and concluded that malnutrition according to the GLIM criteria was a poor prognostic factor for 2-year survival [32]. Our results were comparable with these studies. On the other hand, malnutrition was not a poor prognostic factor for 2-year prognosis in aspiration pneumonia. The mean age of the patients in the study was over 80, which might have affected the result. Furthermore, we presume that there was a shortage of sample size to find poor prognostic factors in the study. Thus, a therapeutic strategy focusing on nutrition status may be useful to improve prognosis in patients with aspiration pneumonia.

This study has several limitations. First, this retrospective study included a small sample of patients with aspiration pneumonia from a single center; therefore, selection bias is of concern and the generalizability of these results to the population of Japan is limited. Second, as most of the patients were super-elderly, some might have died naturally. Third, some possible prognostic factors were not analyzed due to the lack of an adequate sample size. Therefore, further research will be needed to determine whether the score would be helpful in predicting a prognosis in poor-conditioned patients with aspiration pneumonia.

In conclusion, we suggested that the combined score of RR and malnutrition according to the GLIM criteria could predict the prognosis of one- and two-year survival time among the super-elderly with aspiration pneumonia. Further research will be warranted to support these results.

## Figures and Tables

**Table 1:** Comparison of patients’ characteristic and outcomes between survival and death group.

Variables	All Patients	Survival Group	Death Group	*p*-Value
	(*n* = 56)	(*n* = 25)	(*n* = 31)	
Age (mean years ± SD)	86.4 ± 6.5	85.1 ± 6.2	87.4 ± 6.8	0.186
Median age (years, range)	86 (69–105)	85 (69–91)	86 (78–105)	-
Male gender (*n*, %)	25 (45)	9 (36)	16 (51)	0.251
Smoking history (*n*, %)				
Current smoker	3 (5)	2 (8)	1 (3)	0.439
Former smoker	36 (64)	20 (80)	16 (52)	0.027
Never smoker	33 (59)	18 (72)	15 (48)	0.077
Unknown	2 (4)	1 (4)	1 (3)	0.879
Nutritional status (*n*, %)				
Normal	8 (14)	7 (28)	1 (3)	0.001
Malnutrition	48 (86)	18 (72)	30 (97)	0.001
Moderate malnutrition	20 (36)	7 (28)	13 (42)	0.288
Severe malnutrition	28 (50)	11 (44)	17 (55)	0.429
Body mass index (mean kg/m^2^ ± SD)	19.5 ± 3.2	19.7 ± 3.6	19.3 ± 3.0	0.651
Severity of pneumonia (mean points ± SD)				
A-DROP	2.9 ± 0.9	2.8 ± 0.9	3.0 ± 0.9	0.506
CURB-65	2.6 ± 1.0	2.5 ± 1.0	2.6 ± 1.0	0.622
PSI	144.9 ± 28.7	132.2 ± 28.3	155.3 ± 25.1	0.002
I-ROAD *	2.8 ± 0.9	2.6 ± 1.0	3.0 ± 0.8	0.087
SIRS score	1.6 ± 1.2	1.2 ± 1.0	2.0 ± 1.2	0.014
Quick SOFA	1.4 ± 0.7	1.2 ± 0.5	1.5 ± 0.8	0.055
SOFA score	3.2 ± 1.9	2.7 ± 1.7	3.6 ± 2.1	0.081
Underlying diseases (*n*, %)				
Cardiac disease	22 (39)	11 (44)	11 (35)	0.525
Pulmonary diseases	11 (17)	4 (16)	7 (23)	0.546
Diabetes mellitus	12 (21)	3 (12)	9 (29)	0.127
Cerebrovascular diseases	30 (25)	14 (28)	16 (20)	0.749
Paralysis	14 (25)	5 (20)	9 (29)	0.447
Dementia	37 (66)	16 (64)	21 (68)	0.774
Collagen diseases/auto immune disease	5 (9)	3 (12)	2 (6)	0.478
Kidney diseases	9 (16)	4 (16)	5 (16)	0.990
Hemodialysis	5 (9)	5 (20)	0 (0)	0.269
Malignancy	15 (27)	4 (10)	11 (33)	0.105
Gastroesophageal reflux disease	3 (5)	1 (4)	2 (6)	0.692
Proton pump inhibitor user	21 (38)	11 (44)	10 (32)	0.376
Sleeping pill user	13 (23)	6 (24)	7 (23)	0.903
Home oxygen therapy	1 (2)	1 (4)	0 (0)	0.269
CCI score (mean ± SD)	2.6 ± 1.4	2.3 ± 1.4	2.9 ± 1.4	0.107
Higher CCI (≥3) (*n*, %)	28 (50)	10 (40)	18 (58)	0.185
Patients’ condition (*n*, %)				
Respiratory rate (≥22 times/min)	24 (43)	5 (20)	19 (61)	0.003
Loss of consciousness	50 (89)	21 (84)	29 (94)	0.259
Shock vital	8 (14)	3 (12)	5 (16)	0.661
Bacteremia **	17 (34)	6 (32)	11 (35)	1.000
PDR pathogen isolation	14 (25)	6 (24)	8 (26)	0.877
Treatment (*n*, %)				
Mechanical ventilation	1 (2)	0 (0)	1 (3)	0.374
Noninvasive positive pressure ventilation	4 (7)	0 (0)	4 (13)	0.064
Vasopressor	4 (7)	0 (0)	4 (13)	0.064
DNAR order	49 (88)	20 (80)	29 (94)	0.132
ICU admission	0	0	0	-
Inappropriate treatment ***	10 (23)	4 (21)	6 (25)	1.000
Initial treatment failure	2 (4)	1 (4)	1 (3)	0.877
Antibiotics initially used (*n*, %)				
Monotherapy				
Penicillins alone	40 (71)	15 (60)	25 (81)	0.089
Cephalosporins alone	14 (25)	8 (32)	6 (19)	0.277
Carbapenems alone	0	0	0	-
Macrolide alone	0	0	0	-
Fluoroquinolones alone	1 (2)	1 (4)	0	0.261
Combination therapy				
β-lactams plus macrolide	0	0	0	-
β-lactams plus fluoroquinolones	0	0	0	-
Anti-pseudomonal agents	12 (21)	4 (16)	8 (26)	0.374
Anti-MRSA agents	0	0	0	-
Outcomes (mean days ± SD)				
Duration of antibiotics	13.3 ± 10.2	8.6 ± 6.6	17.1 ± 11.2	0.002
Duration of admission	27.1 ± 20.0	23.3 ± 19.0	30.1 ± 20.6	0.212
Survival rate (*n*, %)				
7-day mortality rate	5 (9)	-	-	-
30-day mortality rate	11 (20)	-	-	-
In-hospital mortality rate	6 (11)	-	-	-
One-year mortality rate	31 (55)	-	-	-
Two-year mortality rate	38 (68)	-	-	-

CCI, Charlson Comorbidity Index; DNAR, do not attempt resuscitation; GLIM, Global Leadership Initiative on Malnutrition; ICU, intensive care unit; MRSA, methicillin-resistant *Staphylococcus aureus*; NPPV, non-invasive positive pressure ventilation; PDR, potentially drug resistant; SD, standard deviation; SIRS, systemic inflammatory response syndrome; SOFA, Sequential Organ Failure Assessment. * I-ROAD: A-1, B-2, C-3. ** Blood culture was performed in 216 cases (19 survival and 31 death groups). *** Inappropriate treatment was only assessed in cases who had a result of susceptibility testing.

**Table 2:** Score points of modified R-GLIM score. Modified R-GLIM score consists of respiratory rate (RR) and nutritional status according to the GLIM criteria as follows.

Variables	Scores
Respiratory rate (times/min)	
RR < 22	0
RR ≥ 22	1
Nutritional status	
Normal	0
Moderate malnutrition	1
Severe malnutrition	2

**Table 3:** Correlation between the score points of modified R-GLIM score and prognostic accuracies. Table 2 shows that a modified R-GLIM score of 3 was the appropriate cut-off among patients with aspiration pneumonia.

Modified R-GLIM Score	Sensitivity (%)	Specificity (%)	PPV (%)	NPV (%)	YI
1	96.8	28	62.5	12.5	24.8
2	74.2	46.2	62.2	33.3	20.3
3	41.9	92.3	86.7	43.9	34.2

NPV, negative predictive value; PPV, positive predictive value; YI, Youden Index.

**Table 4:** Univariate and multivariate analyses for 1-year prognosis among aspiration pneumonia patients.

Variables	Univariate Analysis *			Multivariate Analysis		
	Odds Ratio	95% CI	*p*-Value	Hazard Ratio	95% CI	*p*-Value
Respiratory rate ≥ 22	6.3	1.9–21.4	0.002	2.6	1.2–5.6	0.012
Malnutrition	11.7	1.3–102.7	0.017	4.3	0.6–33.6	0.16

CI, confidence interval. * Fisher’s exact test was performed.

**Table 5:** Univariate and multivariate analyses for 2-year prognosis among aspiration pneumonia patients.

Variables	Univariate Analysis *			Multivariate Analysis		
	Odds Ratio	95% CI	*p*-Value	Hazard Ratio	95% CI	*p*-Value
Respiratory rate ≥ 22	6.2	1.5–24.9	0.006	4.5	1.0–2.2	0.042

CI, confidence interval. * Fisher’s exact test was performed.

## Data Availability

All data generated or analyzed during this study are included in this published article.

## Supplementary Materials

The following supporting information can be downloaded at: https://www.mdpi.com/article/10.3390/geriatrics9050118/s1, Table S1: Factors associated with healthcare-associated pneumonia; Table S2: Comparison of microorganisms isolated from 43 sputum samples; Table S3: Comparison of predictive tool for 1-year mortality; Table S4: Comparison of predictive tool for 2-year mortality.
