# Supplementary material for: Modified R-GLIM Score Is a Good Prognostic Tool to Predict a Long-Term Prognosis in Poor Conditioned Elderly Patients with Aspiration Pneumonia, a Pilot Study
Source: Geriatrics (Basel). 2024 Sep 12;9(5):118. doi: 10.3390/geriatrics9050118 (PMC11417869; doi:10.3390/geriatrics9050118)
Supplement: Supplementary file 1 [file geriatrics-09-00118-s001.zip › geriatrics-3086007-supplementary.pdf]

Supplemental Table S1. Factors associated with healthcare-associated pneumonia.

Factors of healthcare-associated	(n, %)
Nursing home residence	30 (54)
Home doctor visit	28 (50)
Prior antibiotic therapy within 90 days	9 (16)
Prior admission history within 90 days	9 (16)
Tube feeding	6 (11)
Hemodialysis	1 (2)
Chemotherapy	1 (2)
Palliative therapy	0
Home oxygen therapy	1 (2)
Immunosuppressive agents use	3 (5)
Poor ADL (PS≥3)	49 (88)

ADL, annual daily life; PS, performance status.

Supplemental Table S2. Comparison of microorganisms isolated from 43 sputum samples.

Microorganisms isolated	All patients (n=56)	Survival group (n=25)	Death group (n=31)	*p*-value
Gram-positive (n, %)				
Streptococcus pneumoniae	1 (2)	0	1 (3)	0.365
Streptococcus anginosus group	2 (4)	0	2 (6)	0.196
Staphylococcus aureus	16 (16)	6 (24)	10 (32)	
MSSA	10 (18)	4 (16)	6 (19)	0.745
MRSA	6 (11)	2 (8)	4 (13)	0.555
Streptococcus agalactiae	7 (13)	4 (16)	3 (10)	0.477
Group G *streptococcus*	0	0	0	-
Corynebacterium spp.	0	0	0	-
Gram-negative (n, %)				
Haemophillus influenzae	1 (2)	0	1 (3)	0.365
Moraxella catahhralis	0	0	0	-
Escherichia coli	5 (9)	0	5 (16)	0.032
Klebsiella pneumoniae	8 (14)	3 (12)	5 (16)	0.661
Pseudomonas aeruginosa	7 (13)	3 (12)	4 (13)	0.919
Enterobacter spp.	2 (4)	0	2 (6)	0.196
Serratia marcescens	2 (4)	2 (8)	0	0.106
Acinetobacter baumanni	0	0	0	-
Stenotrophomonas maltophilia	2 (4)	0	2 (6)	0.196
Normal flora	26 (46)	13 (52)	13 (42)	0.453

MRSA, methicillin-resistant *Staphylococcus aureus*; MSSA, methicillin-susceptible *Staphylococcus aureus*.

Supplemental Table S3. Comparison of predictive tool for 1-year mortality.

Predictive tool	AUC	95%CI	*p*-value
RR-GLIM	0.708	0.573-0.843	0.008
GLIM	0.608	0.455-0.762	0.166
A-DROP	0.529	0.376-0.682	0.711
CURB-65	0.549	0.395-0.703	0.531
PSI	0.735	0.601-0.869	0.003
I-ROAD	0.605	0.456-0.753	0.182
qSOFA	0.635	0.489-0.781	0.085
SOFA	0.63	0.482-0.778	0.098

GLIM, Global Leadership Initiative on Malnutrition; RR, respiratory rate; qSOFA; quick Sequential Organ Failure Assessment; SOFA, Sequential Organ Failure Assessment.

Supplemental Table S4. Comparison of predictive tool for 2-year mortality.

Predictive tool	AUC	95%CI	*p*-value
RR-GLIM	0.669	0.525-0.812	0.043
GLIM	0.564	0.398-0.73	0.44
A-DROP	0.539	0.379-0.699	0.636
CURB-65	0.515	0.349-0.682	0.854
PSI	0.723	0.581-0.865	0.007
I-ROAD	0.586	0.422-0.749	0.305
qSOFA	0.629	0.479-0.778	0.123
SOFA	0.589	0.433-0.745	0.285

GLIM, Global Leadership Initiative on Malnutrition; RR, respiratory rate; qSOFA; quick Sequential Organ Failure Assessment; SOFA, Sequential Organ Failure Assessment.
